# Recurrent Seizure-Like Events in a Toddler With BMPR2-Related Pulmonary Arterial Hypertension

**DOI:** 10.31486/toj.25.0109

**Published:** 2026

**Authors:** Nur Sena Cagatay, Neha Ahluwalia, Aimee Luat

**Affiliations:** ^1^ Department of Pediatrics, Children's Hospital of Michigan, Detroit, MI; ^2^ Division of Neurology, Children's Hospital of Michigan, Detroit, MI; ^3^ Division of Cardiology, Children's Hospital of Michigan, Detroit, MI

**Keywords:** *Bone morphogenetic protein receptors–type II*, *epilepsy*, *pulmonary arterial hypertension*, *seizures*, *syncope*

## Abstract

**Background:**

Seizure-like episodes are common in children, but many spells are not true epileptic seizures. Cardiac or pulmonary conditions such as pulmonary arterial hypertension can be the cause of the seizure-like episodes, particularly when the events persist despite administration of antiseizure medications.

**Case Report:**

A 2-year-old male presented with recurrent seizure-like episodes that persisted despite administration of 2 antiseizure medications. Spells consisted of a sudden collapse followed by body jerking. Electroencephalogram and brain imaging were normal. Given the atypical features and lack of medication response, a cardiology evaluation was pursued. Echocardiography revealed severe pulmonary arterial hypertension with markedly elevated right heart pressures. Genetic testing confirmed the presence of a pathogenic bone morphogenetic protein receptor type 2 (BMPR2) gene. Despite initiation of sildenafil, bosentan, and prostacyclin therapy, the patient's condition worsened with recurrent syncopal and cyanotic episodes. He suffered a cardiac arrest requiring prolonged resuscitation and extracorporeal membrane oxygenation support, complicated by hypoxic-ischemic encephalopathy and multiorgan failure. Following discussions with the family, care was redirected to comfort measures, and the patient died.

**Conclusion:**

This case illustrates how pulmonary arterial hypertension may mimic drug-resistant epilepsy. Rather than seizures, the patient's events were syncope resulting from decreased cerebral perfusion caused by low cardiac output. BMPR2 mutations are the most common genetic cause of heritable pulmonary arterial hypertension and are associated with earlier onset, more severe disease, and poor response to therapy compared to pulmonary arterial hypertension without BMPR2 mutations. To our knowledge, this case is the first report of BMPR2-related pulmonary arterial hypertension initially presenting as seizure-like episodes in a child. Children with atypical presentations should be evaluated for cardiopulmonary causes. Early recognition of pulmonary arterial hypertension is essential, as delayed diagnosis limits treatment opportunities.

## INTRODUCTION

Epileptic seizures are common in children, but paroxysmal events in children may not be true epileptic seizures; instead, they may be mimics such as syncopal episodes from cardiac and noncardiac etiologies, breath-holding spells, or psychogenic events.^1,2^

We report the case of a toddler whose recurrent events initially prompted a neurologic workup and diagnosis of epileptic seizure but who was ultimately diagnosed with severe pathogenic bone morphogenetic protein receptor type 2 (BMPR2)–related pulmonary arterial hypertension. To our knowledge, this case is the first report of a pediatric patient with a BMPR2 pathogenic variant whose initial presentation mimicked drug-resistant epilepsy.

## CASE REPORT

A previously healthy 2-year-old male presented to the emergency department (ED) with recurrent spells of sudden distress, limpness, and convulsive events (described as whole-body rhythmic shaking, eye-rolling, and bluish coloration around the mouth), followed by unresponsiveness. Brain computed tomography (CT) and routine electroencephalogram (EEG) were normal. Family history revealed a cousin with a history of febrile seizures. Birth and developmental history were normal. The patient was seen in the neurology clinic 5 days following the ED visit. The family described 3 similar seizure-like episodes during the 1 month prior to the ED visit. Given the recurrent unprovoked convulsions, a clinical diagnosis of epilepsy was suspected, and the patient was started on levetiracetam 25 mg/kg/d. Despite maximizing levetiracetam to 40 mg/kg/d, the patient had 2 more seizure-like episodes during the following 2 months, prompting 2 ED visits. Clobazam up to 1 mg/kg/d was added to his treatment regimen. The patient was referred to the epilepsy clinic.

At the epilepsy clinic, the patient's mother described 3 distinct types of spells: (1) generalized shaking with eye-rolling and postictal sleepiness; (2) sudden limpness and unresponsiveness, sometimes preceded by crying; and (3) brief staring spells with glassy eyes and rapid recovery. Given the patient's atypical features and lack of response to antiseizure medications, cardiology evaluation was pursued within 2 months after initial presentation. Echocardiography demonstrated a moderately to severely dilated right ventricle (Figure) with mildly depressed systolic function. Estimated right ventricular systolic pressure was at least 120 mm Hg, while mean pulmonary arterial pressure was at least 30 mm Hg. Left ventricular size and systolic function were normal; blood pressure was 101/63 mm Hg during the echocardiogram. The patient was diagnosed with severe pulmonary arterial hypertension and was immediately admitted. Further workup included CT angiography of the thorax and abdomen that showed moderately dilated right heart chambers with a thickened ventricular wall and pulmonary edema.

**Figure.**
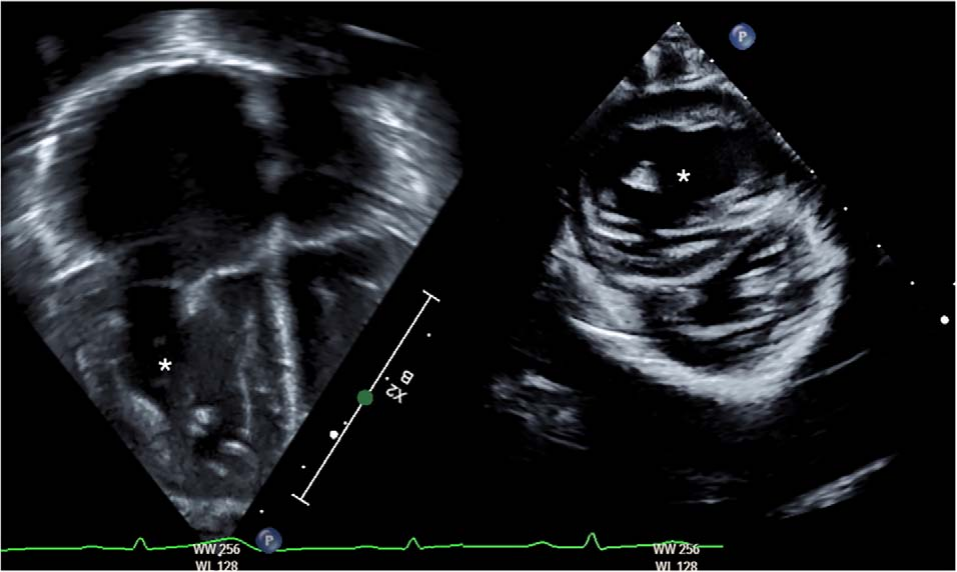
Transthoracic echocardiogram, apical 4-chamber view, shows a markedly dilated and hypertrophied right ventricle. The asterisks (*) denote the right ventricle.

Given the severity of the pulmonary arterial hypertension, the patient was started on triple therapy consisting of sildenafil (1 mg/kg twice daily), bosentan (2.5 mg/kg twice daily), and selexipag (300 μg twice daily). Genetic testing identified a heterozygous pathogenic variant in the BMPR2 gene, confirming autosomal dominant pulmonary arterial hypertension. Later maternal testing revealed the same variant, but the patient's mother had no clinical evidence of pulmonary arterial hypertension which was attributed to incomplete penetrance.

The patient was gradually weaned off antiseizure medications, and no seizure-like episodes occurred on the new regimen while he was an inpatient. After 3 weeks in the hospital, the patient was discharged home on the same triple therapy for pulmonary arterial hypertension with plans for outpatient follow-up.

However, after 4 months, the patient again began having similar seizure-like events and was admitted for syncopal and cyanotic episodes. Cardiac catheterization revealed markedly elevated pulmonary artery pressure (70 mm Hg) with a pulmonary vascular resistance index of 13.2 Wood units. The patient underwent balloon atrial septostomy to decompress the failing right ventricle, to improve left ventricular preload, and to augment cardiac output. He was started on a subcutaneous treprostinil infusion, with dose titration to 28 ng/kg/min, as well as low-dose oral aspirin (81 mg daily). After 1 week in the hospital, the patient was discharged in stable condition on subcutaneous treprostinil and aspirin therapy, in addition to oral sildenafil and bosentan. Postdischarge, the continuous treprostinil infusion was administered via a subcutaneous pump.

Despite the aggressive pulmonary hypertension management with triple therapy, the patient presented 1 week later to the ED with worsening fatigue, poor appetite, and syncope. His oxygen saturation was 75%. Two days after admission to the cardiac intensive care unit (ICU), the patient's hypoxia decreased to the 50s, and he was intubated. Hemodynamics continued to deteriorate with hypotension (blood pressure of 61/33 mm Hg) and bradycardia (heart rate of 16 beats per minute). Cardiac arrest occurred on day 3 of admission, requiring 65 minutes of cardiopulmonary resuscitation before extracorporeal membrane oxygenation (ECMO) cannulation. The patient's course was complicated by hypoxic-ischemic encephalopathy, severe biventricular failure, and multiorgan dysfunction. After multidisciplinary and family discussions, the decision was made to transition the patient to comfort care. On day 9 of his cardiac ICU admission, he was compassionately decannulated from ECMO and died.

## DISCUSSION

Pediatric patients who present with a seizure-like episode should undergo a thorough evaluation that includes a comprehensive history, neurologic examination, EEG, and targeted laboratory and imaging studies. No single clinical symptom reliably distinguishes epileptic seizures from nonepileptic events, making careful history-taking, thorough examination, and close follow-up essential.

Our case illustrates how cardiopulmonary disorders can mimic epileptic seizures. The child's episodes were initially treated as epilepsy, but he had no clinical response to increasing antiseizure medication dosages. Key features that suggested a nonepileptic cause included atypical episodes with multiple seizure semiology, rapid recurrence of symptoms despite antiseizure medications, and normal neurologic workup, including EEG and imaging findings.

In cases of pulmonary arterial hypertension, the seizure-like episodes may actually represent hypoxic events or syncope resulting from decreased cerebral perfusion caused by low cardiac output rather than true epileptic seizures.^2^ This etiology is supported by case reports of children with medically resistant nocturnal events that were initially misdiagnosed as suspected epilepsy but later found to be idiopathic pulmonary arterial hypertension following a syncopal event.^3,4^

The clinical presentation of pediatric pulmonary arterial hypertension is often subtle and can include unexplained syncope, fatigue, or seizure-like episodes, particularly in the absence of overt cardiopulmonary symptoms. The literature highlights that symptoms of pulmonary arterial hypertension in children are frequently misleading, resulting in delayed diagnosis until classic features or complications emerge.^1,5^ In our case, echocardiography ultimately revealed severe pulmonary hypertension that was genetically confirmed as being related to BMPR2-associated pulmonary arterial hypertension. BMPR2-related pulmonary arterial hypertension in pediatric patients is a heritable form of the condition caused by pathogenic variants in the BMPR2 gene.^6^

Pathogenic variants in the BMPR2 gene are a primary genetic cause of pulmonary arterial hypertension,^6,7^ which is identified in approximately 70% of familial cases^6,8^ and approximately 25% of idiopathic cases.^6,9^ BMPR2 pathogenic variants are associated with more severe disease characteristics compared to pulmonary arterial hypertension without BMPR2 mutations, including earlier onset, elevated pulmonary pressures, lower cardiac index, less likelihood to respond to treatment, and an increased risk of all-cause mortality, particularly in patients diagnosed at a younger age.^7^

Given the central role of BMPR2 variants in heritable pulmonary arterial hypertension, genetic testing should be considered in pediatric patients with unexplained disease. Careful assessment of family history is essential, as genetic testing provides diagnostic clarification and enables effective family screening, identification of at-risk relatives, and accurate counseling regarding recurrence risk. The familial form has autosomal dominant inheritance; however, genetic counseling should be performed before genetic testing to address the complex issues of incomplete penetrance.^5^ The pathobiology of BMPR2-related pulmonary arterial hypertension involves disrupted signaling in the transforming growth factor-β superfamily, leading to abnormal pulmonary vascular remodeling and clinical progression to right heart failure.^10^ The American Heart Association and the American Thoracic Society recommend genetic testing for BMPR2 and related genes in pediatric patients with pulmonary arterial hypertension to aid in risk stratification, family counseling, and management.^5^

Our review of the literature showed no prior reports of BMPR2-related pulmonary hypertension initially presenting as seizure-like episodes or drug-resistant epilepsy. This case, therefore, expands the clinical spectrum of BMPR2-associated disease and highlights the importance of considering cardiopulmonary causes when children with presumed epilepsy show atypical features. Early recognition of pulmonary arterial hypertension is crucial, as targeted therapies such as endothelin receptor antagonists, phosphodiesterase type 5 inhibitors, and intravenous or subcutaneous prostacyclin analogs can improve survival, although the prognosis for pediatric patients with BMPR2-related pulmonary arterial hypertension remains guarded.^11,12^

## CONCLUSION

Children who experience recurrent seizure-like episodes and present with various semiology, a normal neurologic workup, and a lack of response to appropriate antiseizure medications should be evaluated for cardiopulmonary causes. This case highlights the diagnostic challenges and poor prognosis associated with BMPR2-related pulmonary arterial hypertension that can present as seizure-like symptoms in early childhood. Clinicians should maintain a high level of suspicion for cardiac or pulmonary issues in children experiencing “seizures” that do not respond to multiple antiseizure medications.

## Notes

*This article meets the Accreditation Council for Graduate Medical Education and the American Board of Medical Specialties Maintenance of Certification competencies for Patient Care and Medical Knowledge.*
